# Antisense Oligonucleotide-Based Therapy for Neuromuscular Disease

**DOI:** 10.3390/molecules22040563

**Published:** 2017-04-05

**Authors:** Valentina Sardone, Haiyan Zhou, Francesco Muntoni, Alessandra Ferlini, Maria Sofia Falzarano

**Affiliations:** 1 Dubowitz Neuromuscular Centre, Molecular Neurosciences Section, Developmental Neuroscience Programme, UCL Great Ormond Street Institute of Child Health, London WC1N 1EH, UK; v.sardone@ucl.ac.uk (V.S.); haiyan.zhou@ucl.ac.uk (H.Z.); 2 MRC Centre for Neuromuscular Diseases, UCL Institute of Neurology, London WC1N 3BG, UK; 3 UOL Medical Genetics, University of Ferrara, Ferrara 44121, Italy; flzmsf@unife.it

**Keywords:** Duchenne Muscular Dystrophy, Spinal Muscular Atrophy, antisense oligonucleotides, clinical trials, oligonucleotides delivery

## Abstract

Neuromuscular disorders such as Duchenne Muscular Dystrophy and Spinal Muscular Atrophy are neurodegenerative genetic diseases characterized primarily by muscle weakness and wasting. Until recently there were no effective therapies for these conditions, but antisense oligonucleotides, a new class of synthetic single stranded molecules of nucleic acids, have demonstrated promising experimental results and are at different stages of regulatory approval. The antisense oligonucleotides can modulate the protein expression via targeting hnRNAs or mRNAs and inducing interference with splicing, mRNA degradation, or arrest of translation, finally, resulting in rescue or reduction of the target protein expression. Different classes of antisense oligonucleotides are being tested in several clinical trials, and limitations of their clinical efficacy and toxicity have been reported for some of these compounds, while more encouraging results have supported the development of others. New generation antisense oligonucleotides are also being tested in preclinical models together with specific delivery systems that could allow some of the limitations of current antisense oligonucleotides to be overcome, to improve the cell penetration, to achieve more robust target engagement, and hopefully also be associated with acceptable toxicity. This review article describes the chemical properties and molecular mechanisms of action of the antisense oligonucleotides and the therapeutic implications these compounds have in neuromuscular diseases. Current strategies and carrier systems available for the oligonucleotides delivery will be also described to provide an overview on the past, present and future of these appealing molecules.

## 1. Introduction

The availability of novel drugs in genetics medicine can offer new opportunities for treating conditions for which there is currently no therapeutic strategy. This lack of therapies is dramatic in rare genetic diseases and has instigated many researches aiming at identifying new molecules.

Although proteins are the most common target of already approved drugs [1], research has also intensively focused on the discovery of new biologically active molecules, which target the nucleic acids, as RNA or DNA [2].

Among these new compounds, antisense oligonucleotides (ASOs) have been recognized for the treatment of neurodegenerative disorders [3]. The use of ASOs, that target specifically hnRNAs or mRNAs, allows interference with the splicing mechanism, or regulation of protein translation, or RNA/protein binding. In this way the ASO molecules can alter the expression of specific genes with a therapeutic application for pathologies that are not treatable with other known drugs [4,5].

Earlier studies in the neuromuscular field reported interesting, although somewhat conflicting results from experimental clinical trials where two classes of ASOs (a 2′-*O*-methyl and a phosphorodiamidate morpholino) were used to rescue the dystrophin protein synthesis in Duchenne Muscular Dystrophy (DMD) via splicing modulation [6].

Very recently, Spinraza (previously known as Nusinersen), an *O*-(2-methoxyethyl) modified ASO administered intrathecally, has moved from phase I, to phase II and III studies for infants and children with Spinal Muscular Atrophy (SMA), with very encouraging tolerability and highly significant clinical benefit for treated patients [7,8].

Several clinical trials based on ASO therapies reported excellent results also for other diseases such as for the treatment of hepatitis C virus (HCV) infection targeting microRNA with Miraversen, a locked nucleic acid-modified DNA phosphorothioate oligonucleotide linked to a *N*-acetylgalactosamine carbohydrate structure [9].

This review will provide an update on the different antisense chemistries developed and the delivery systems currently available, and will describe their clinical applications in the neuromuscular fields.

### 1.1. ASO Therapy in Duchenne Muscular Dystrophy

Duchenne Muscular Dystrophy (DMD) is a rare and progressive neuromuscular disorder affecting approximately 1 in 5000 newborn boys. The disease is characterized by progressive muscle-wasting and weakness leading to loss of ambulation by the mid-adolescent years [10]. DMD is due to mutations in the dystrophin-encoding *DMD* gene. According to the Leiden muscular dystrophy database (www.dmd.nl), exons deletions or duplications are present in about the 80% of DMD patients, whereas 20% carry small mutations [11]. ASOs have been developed to induce exon skipping and restore the transcript reading frame, which is disrupted in DMD cases carrying out of frame mutations. ASOs promote the translation of an internally deleted dystrophin protein mimicking what occurs in the milder allelic Becker Muscular Dystrophy (BMD) [12]. The rescue of a shorter protein should slow down muscle fiber degeneration and ameliorate disease progression. Since late 1990s, splice switching ASOs have been extensively tested on cellular models (human and not human) [13,14] and on animal models (including mouse and dog, for review [15]). The majority of DMD mutations cluster into hot spots between exon 43 and 53 meaning that 69% of patients with deletions can be treated by single exon skipping, rising to 90% if multi-exon skipping can be achieved [16].

In particular, mutations amendable for exon 51 skipping are present in the 13% of DMD boys, representing the biggest group of patients potentially treatable with exon skipping approach: for this reason ASO therapy was firstly developed to correct this group of DMD patients.

### 1.2. ASO Therapy in Spinal Muscular Atrophy

Spinal Muscular Atrophy (SMA) is an autosomal recessive disease, which is characterized by progressive symmetrical muscular atrophy and weakness as a result of degeneration of motor neurons in spinal cord. It is the most common genetic cause of infant mortality and affects approximately 1 in 6000 to 1 in 10,000 live births with a carrier frequency of 1 in 35~50 in the general population [17,18]. The genetic defect in SMA patients is the loss of survival of motor neuron (SMN) protein, caused in 95% of cases by homozygous deletions of the Survival of Motor Neuron 1 (*SMN1*) gene [19]. The SMN locus is part of a genomic inverted duplication on human chromosome 5 which results in the paralogue gene, *SMN2*. *SMN2* is intact in all SMA patients. Furthermore, *SMN2* copy number is variable in the general population, and in SMA, there is an inverse correlation between the number of copies of *SMN2* and the severity of the disease [20,21]. The *SMN1* and *SMN2* genes differ by several exonic and intronic SNPs although no amino acid substitution is induced by these variants. Among them, a single-nucleotide cytosine (C) to thymidine (T) transition in *SMN2* at position 6 of exon 7, significantly disrupts the splicing of this exon and leads to the predominant (~90%) exon 7 skipping [22,23,24,25]. The remaining 10% of its transcripts contains exon 7, however, the low abundance of the resulting protein is not sufficient to fully compensate for the loss of the *SMN1* gene.

SMA is currently incurable; however, significant progress in the development of therapeutic strategies has been achieved. Since the fundamental defect in SMA is the deficiency of SMN protein, most of the strategies are focusing on increasing the expression of SMN protein. These include the following: (i) ASO therapy for redirecting the splicing of exon 7 in *SMN2* to increase the full-length *SMN2* transcript [26,27,28,29,30]; (ii) gene therapy of increasing functional SMN protein by introducing exogenous *SMN1* gene using viral vectors [31,32,33,34]; and (iii) small-molecule drugs to stimulate promoter activity [35,36,37,38]. In addition, other strategies are being used including neuroprotection with small compounds and transplantation of neuron stem cells [39,40,41].

## 2. Cellular Uptake Mechanism of Antisense Oligonucleotides

Cellular and extra cellular nucleases are capable of degrading rapidly unmodified DNA and RNA ASOs and for this reason these molecules could not been used without modification in vivo. An efficient ASO should have high specificity and binding to its target molecule without off-target effects. ASOs cross the cellular membrane exploiting different internalization methods based on their chemical structure and on the membrane peculiarities of the target cells. It has been demonstrated that ASOs could enter into cells by diffusion [42], even though the most studied and reported ASO mediated uptake is via endocytosis [7].

### 2.1. ASO Internalization Process Mediated by Endocytosis

ASO internalization process is mediated by different and complex pathways. Clathrin-mediated endocytosis is one of the most common internalization processes occurring after the ligand-cell surface receptor interaction [43]. Caveolin 1 is another protein which could mediate small molecules internalization and it is normally highly expressed in cholesterol-rich cellular membranes [44]. There are also clathrin- and caveolin-independent pathways which require the involvement of other proteins such as the flotillins, the CLIC/GEEC pathway and protein kinases belonging to the PAK family [45]. 

### 2.2. ASO Uptake by Cell-Penetrating Peptides

Discovered in the late 1980s, cell penetrating peptides (CPPs) are another mechanism to enhance ASO internalization. CPPs are small peptides, typically less than 30 amino acids, capable of traversing the cell membrane [46]. The first CPP to be discovered is a sequence of 9 amino acids expressed in the trans-activactor protein (TAT) [47,48]. Around the same period, another peptide region of 60 amino acids was discovered from the antennapedia homeobox protein of Drosophila [49]. This peptide sequence, subsequently named Penetratin, was able to cross the cellular membrane of differentiated neurons [49]. CPPs linked to ASOs are under investigations in animal models of several neuromuscular disorders [50] and recently this delivery method has shown promising results targeting efficiently also the central nervous system after systemic delivery [51]. The majority of CPPs have a high cationic charge and this makes their conjugation with negatively charged ASO practically impossible [52]. For this reason, the most successful experiments of CPP conjugation have been with neutrally charged ASOs such as peptide nucleic acid (PNA) or phosphorodiamidate morpholino (PMO) [46]. Ivanova and colleagues demonstrated that CPPs conjugated to a 20-mer PNA for targeting dystrophin correction in exon 23 in the DMD mouse model (*mdx*) showed higher exon skipping activity in differentiated mdx mouse myotubes and higher number of dystrophin-positive fibres when injected in the tibilias anterior of *mdx* mice compared to naked PNA [53]. CPP-ASO conjugates are targeting skeletal muscles and are also able to support dystrophin restoration in the heart of *mdx* CPP-ASO treated mice [54], although the toxcity observed for some of these compounds in the preclinical models currently precludes their use in clinical trials [54].

### 2.3. Limiting Factors for ASOs Uptake

Usually, cellular models used to study ASO uptake do not reflect the *in vivo* delivery complexity. Many factors should be considered after ASO administration such as drug half-life, clearance rates, pharmacodynamic and pharmacokinetic properties. ASOs should be administrated at a different range of doses depending on the type of ASO and on the target tissue. Furthermore, some types of ASO accumulate in liver, kidney and intestine requiring high doses of administration to reach relevant pharmacological concentration in any other target tissue. Nevertheless, when an ASO successfully passes the in vitro and in vivo validation testing and approaches clinical applications, manufacturing feasibility and cost effectiveness should also be considered. Indeed, ASO needs to be synthetized on large scale and under Good Manufacturing Practice (GMP).

## 3. Different Chemistries of Antisense Oligonucleotides

Unmodified DNA and RNA molecules cannot be used, not only because they are susceptible to intra and extracellular nucleases. Nucleic acids with no modifications bind weakly to plasma proteins and are very rapidly filtered by the kidneys and excreted into the urine.

### 3.1. Phosphorothioate ASOs

**Figure 1:**
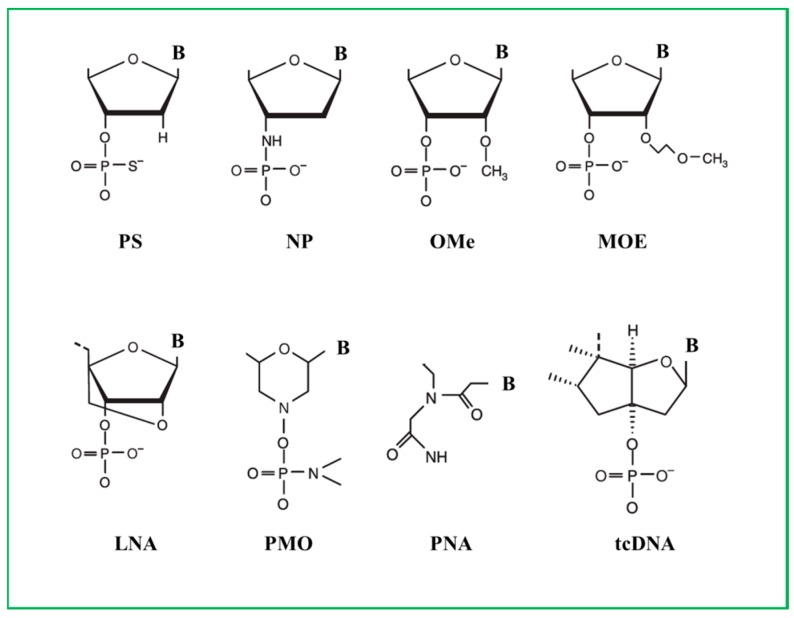
Chemical modifications of ASO backbone. PS, phosphorotioate; NP, N3′-P5′ phosphoroamidate; OMe, 2′-*O*-methyl; MOE, 2′-*O*-methoxy-ethyl; LNA, locked nucleic acid; PMO, phosphoroamidate morpholino; PNA, peptide nucleic acid; tcDNA, tricyclo DNA.

The first generation of ASO drug synthetized and clinically tested was on a phosphorothioate (PS) backbone. These molecules gain nuclease resistance by the substitution of the non-bridging phosphate oxygen with a sulphur atom (Figure 1) [55]. PS ASOs are able to induce RNase H cleavage promoting target transcript degradation. These drugs show increased binding to plasma proteins which results in prolongation of their half-life [56].

Another backbone modification is the substitution of the 3′oxygen in the deoxyribose ring with a 3′amino atom [56]. These compounds, called N3′ P5′ phosphoramidate oligodeoxynucleotides, exhibit high stability to nucleolytic degradation and increased target binding, but do not activate RNase H.

### 3.2. 2′-O-Methyl and 2’-O-Methoxyethyl ASOs

Core backbone modifications of the PS structure increase stability as it occurs in the 2′-*O*-methly (2′MeO) and in the 2′-*O*-methoxyethyl (2′MOE) ASOs (Figure 1). These chemical modifications make the ASOs more resistance to nuclease and even more specific to their binding to RNA [56]. Compared to the phosphodiester, these molecules could be potentially more cytotoxic due to their chemical structure and could generate immunogenic responses [57].

### 3.3. Locked Nuclei Acid ASOs

Locked nuclei acid (LNA) compounds were generated as constrained analog of the 2’*O*-methyl-RNA where the 2′ residue is tethered with the carbon atom located in position 4′ (Figure 1). This bridge bound results in a 3′-endo-conformation, which reduces the conformational flexibility of the ribose. They do not support RNase H activity but with gapmer addition, they could induce transcripts down regulation mediated by RNase H [58]. LNAs are nuclease resistant, non-toxic and negatively charged. Co-administration with cation lipid polymers increases membrane crossing rate and upturns in delivery efficacy. LNAs were successfully tested to induce exon 46 skipping in DMD patient cells carrying an exon 45 deletion [59].

### 3.4. Phosphorodiamidate Morpholino ASOs

Chemical modifications can be performed also in the sugar backbone with the generation of phosphorodiamidate compounds. Phosphorodiamidate morpholinos (PMO) belong to this family and they were synthetized with a morpholino ring at the place of the furanose ring. The nitrogen morpholino atom is connected to the hydroxyl group at the 3′ residue by a phosphorodiamidate linkage (Figure 1). Morpholino oligomers were conceived by James E. Summerton (Gene Tools) and developed in collaboration with a biotech company (Antivirals, renamed AVI-BioPharma and more recently Sarepta Therapeutics). To interact with the target PMO should be long at least 15 nucleotides; this increases their specificity and decreases their off-target effects [56]. They are non-toxic, neutrally charged and highly soluble in water. PMOs block protein translation but do not activate transcript degradation mediated by RNase H. Due to their characteristics they are widely used for translation blocking or as splice switching antisense [60].

### 3.5. Peptide Nucleic Acids ASOs

Peptide nucleic acids (PNAs) belong to another family of ASOs generated by structural change to the nucleic acid backbone. The sugar phosphate backbone is replaced by a peptide, which gives the molecule the ability to resist to the cellular and extra cellular nucleases and proteases. Compared to 2′-OMePS backbone compounds, PNAs have a highly increased affinity for DNA and RNA [61]. Due to their cationic nature, they are poorly soluble in water and so very difficult to be transfected. Lysine additions to the peptide sequence could increase solubility [62]. As previously discussed in Section 2.2, PNA conjugated to CPPs demonstrated higher skipping efficiency compared to the naked PNA [53].

### 3.6. Tricyclo-DNA ASOs

A new class of compounds has been recently developed: the tricyclo-DNA (tcDNA) ASOs. tcDNA could be synthetized by standard phosphoramite chemistry and are resistant to nuclease activity [63]. Chemically, tc-DNA derives from a DNA backbone with the addition of bound between C (5′) and C (3′) (Figure 1). In a recent paper Goyenvalle and collaborators showed that tc-DNA ASO was able to improve cardio-respiratory and neuromuscular functions in two DMD mouse models: the *mdx* mouse model, and the *Utrn* (encoding utrophin)-*Dmd* double knockout mouse model which develops even more severe muscular dystrophy than the *mdx* model [64,65,66]. These authors showed that tc-DNA were 3–4 times more efficient to induce exon skipping compared to the same dose administrated of 2′-*O*-Me or PMO ASOs. Interestingly, they also demonstrated that tc-DNA ASOs were able to cross the brain blood barrier (BBB) and to correct also dystrophin deficiency in *mdx* mouse hearts. If these ASOs will be demonstrated to be safe following the preclinical toxicology development, they could potentially be attractive for treating not only DMD neuromuscular symptoms, but also the associated cardiomyopathy and behavioral abnormalities currently frequently associated with the disorder [12].

## 4. Mechanism of Action of ASOs

ASOs can modulate gene expression acting in different ways based on their chemistry and the final target.

One of the mechanisms is the RNase H-mediated degradation by a selective cleavage of the pre-mRNA. Since ASOs have 2′ modifications and the RNase H requires a free 2′-*O*, gapmer antisense nucleotides should be inserted. Gapmers consist of a central region that supports RNase H activity with flanking chemically modified ends that protect the ASO from nucleases [7,67].

**Figure 2:**
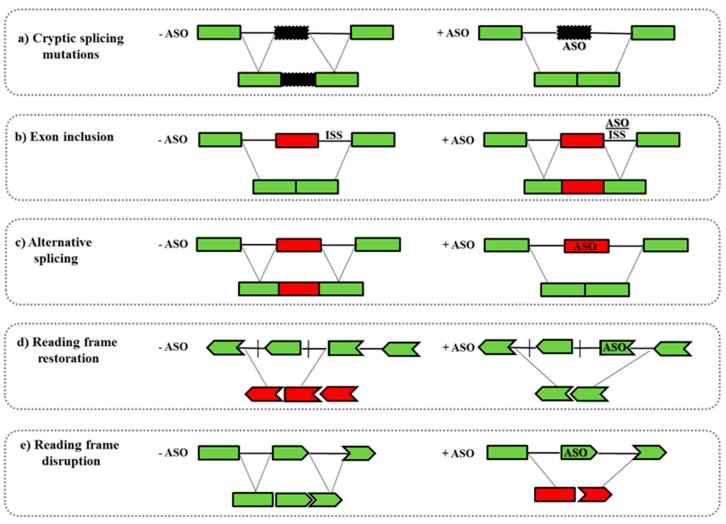
Antisense-mediated exon skipping mechanisms. (**a**) Cryptic splicing mutations induce the inclusion of an aberrant exon (black box) into the mature transcript and the ASO can skip the cryptic exon restoring the normal transcript; (**b**) exon inclusion can be induced by ASO that, targeting the intronic splicing silencers (ISS) generated by the mutation, restores the correct exon inclusion (red box); (**c**) ASO can be used for switching between alternative splicing isoforms; (**d**) ASO can support exon skipping restoring the correct reading frame; (**e**) ASO can induce the translation of an internally deleted protein or can generate reading frame disruptions with consequent partially or complete transcript knockdown.

- Another well-studied application is the splicing modulation of the nuclear pre-mRNA. ASOs can target specific regions as 5′/3′ splice junctions or exonic/intronic splicing enhancer/silencer sites (ESEs or ISEs, ESSs or ISSs) leading to the skipping/inclusion of an exon. This strategy can be used to: (i) restore the mRNA reading frame by exon skipping in diseases such as DMD in which frame-shift deletions or non-sense mutations cause the functional protein loss; (ii) promote the inclusion of exons, as occurs in SMA where ASOs induce the inclusion of the exon 7 in the *SMN2* gene; (iii) introduce an out-of-frame deletion for reducing protein expression, as in Alzheimer disease or in Amyotrophic Lateral Sclerosis (Figure 2) [67].
- ASOs can also target microRNA (miRNAs) that are involved in many disease mechanisms and could be used as therapeutic tools. miRNAs are able to silence the target mRNA as well as influencing multiple mRNA expression profiles. Targeting miRNA regulation by using ASOs could be very effective in clinical interventions, as has been demonstrated for hepatitis C infection (antimiR122), breast cancer (anti-miR221), and brain tumors (anti-miR155) [68,69]. Catapano and colleagues showed that the systemic treatment of SMA mice with a single dose of oligomer PMO25 is able to reverse the altered miR-132 levels in spinal cord, muscle, and serum [70].
- Recent studies on Facioscapulohumeral Muscular Dystrophy (FSHD) suggest that targeting mRNA polyadenylation signal and/or cleavage site of Double homeobox 4 (*DUX4*) transcription factor, by using ASOs, can be a potential and promising strategy to down-regulate DUX4 expression [71,72].
- ASOs can also interfere in the binding between proteins and pathogenic RNA species. Myotonic dystrophy type 1 (DM1) is a neuromuscular disease caused by expanded CUG repeats in the 3′-untranslated region of the DM protein kinase (*DMPK*) transcript [73]. A morpholino ASO has been developed, CAG25, and it is able to form a stable RNA-morpholino heteroduplex with the pathogenic *DMPK* transcripts carrying the CUG repeats. In this way, CAG25 blocks the interaction of these abnormal RNA species with other proteins such as muscleblind-like 1 (MBNL1), which has a fundamental role in the control of the splicing machinery [74]. Mulders and colleagues developed a 2′-*O*-methyl-phosphorothioate modified (CAG)7 ASO that silences the toxic *DMPK* transcript and induce a normalizing effect on aberrant pre-mRNA splicing [75].

## 5. Pre-Clinical and Clinical Approaches of ASOs in DMD

Different chemical splice switching ASOs have been developed and are herein described by their chemical properties and outcome in pre-clinical and clinical applications. A summary of clinical trials where ASOs have been used to treat DMD patient cohorts is reported in Table 1.

### 5.1. Phosphorothioate ASOs

Phospohorothioate ASOs were the first compounds developed and successfully administrated for treating cytomegalovirus-induced retinitis in AIDS patients [76].

A 31-mer PS was developed and firstly tested in 2006 in a single DMD patient carrying deletion in exon 10 of the dystrophin gene. They demonstrated that the drug was safe and could be administrated by intramuscular infusion. Dystrophin restoration was observed both at transcriptional and translational levels by PCR and immunofluorescence techniques, respectively [77].

### 5.2. 2′OMe ASOs

In 2007, Prosensa developed a 2′OMePS ASO for treating DMD patients potentially treatable by exon 51 skipping. The drug, originally named PRO051, eventually renamed Drisapersen, was administrated by intra muscular injection into the tibialis anterior muscle of 4 DMD patients. Following one single administration, protein quantification was assessed by Western blot showing a restoration from 17% to 35% of normal levels when dystrophin expression values were normalized to laminin A2 levels. After ASO treatment dystrophin expressing fibers ranged between 64% and 97% [78]. This preliminary study laid down the foundation for an open-label dose escalation phase I-IIa trial where Drisapersen was administrated in 12 patients weekly by abdominal subcutaneous injections for 5 weeks. Different ranging doses (0.5, 2.0, 4.0, and 6.0 mg/kg) were tested and after safety assessment all the patients received the 6.0 mg/kg dose regimen for additional 12 weeks [79]. Tibialis anterior muscle biopsy was performed at baseline and after 2 weeks of the last dose of Drisapersen in only 3 patients corresponding to the cohort treated with 0.5 mg/kg of the drug. The other cohorts of patients (2, 4 and 6 mg/kg) underwent muscle biopsy procedure after 2 and 7 weeks of treatment from the last dose received. The results of this study were interesting with an increase of dystrophin positive fibers and overall dystrophin protein expression (quantified by Western blot) [79]. Also the 6-minute walk test (6MWT) reflected the clinical benefits of Drisapersen with a mean (±SD) improvement of 35.2 ± 28.7 m after treatment. However, these data on dystrophin restoration need to be interpreted carefully due to the fact that very few patients (*n* = 3) who received the predicted therapeutic doses had a baseline muscle biopsy, making challenging to demonstrate the production of low levels of dystrophin against the background of low level of residual dystrophin expression, an invariable feature in the DMD boys [12]. Recently the 188 week open label extension of the same DMD patients was reported [79]. Indeed, the authors acknowledged it had not been possible to determine the levels of dystrophin induced by the Drisapersen ASO due to the lack of a baseline muscle biopsy. Nevertheless the clinical assessment of these patients demonstrated a very encouraging improvement of the 6 min walk test values compared to natural history controls, suggesting a drug effect on slowing down disease progression [80].

A separate phase II study, placebo controlled, was conducted in order to assess safety and efficacy of two Drisapersen dose regimens (continuous and intermittent) for a period of 48 weeks [81].

Muscle biopsies were performed after 1 week and 45 weeks of treatment. Biological assays such as RT-PCR, Western blot and immunofluorescence showed a small pharmacodynamics effect (dystrophin rescue) associated with the Drisapersen regimen. Statistically significant improvement in motor functions were detected in the patients treated with continuous regimen after 24 weeks of treatment, and while the differences were not significant at 48 weeks, a positive trend in favour of Drisapersen was observed at this time-point [81].

Subsequently, 186 DMD patients were enrolled in a Drisarpersen phase III clinical trial. The results of this study however failed to meet the primary clinical endpoint (improvement in the 6 MWT in the DMD treated cohort compared to the placebo) [82]. A possible explanation of the difference between this and previous studies relates to the considerably more advance disease status of DMD boys recruited in this latter phase III compared to the previous phase II studies. As in DMD there is a progressive loss of muscle mass, it is conceivable that a therapy which aims to slow down disease progression might fail to demonstrate clinical efficacy if the disease progress and the loss of muscle mass is too far advanced.

In November 2014, BIOMARIN, an US biopharmaceutical company developing therapies for rare diseases, acquired Prosensa with the goal to pursue the development of the ASO asset and obtain the regulatory authorities approval (both from the Food and Drug Administration (FDA) and the European Medical agency (EMA)) [83]. 

**Figure 3:**
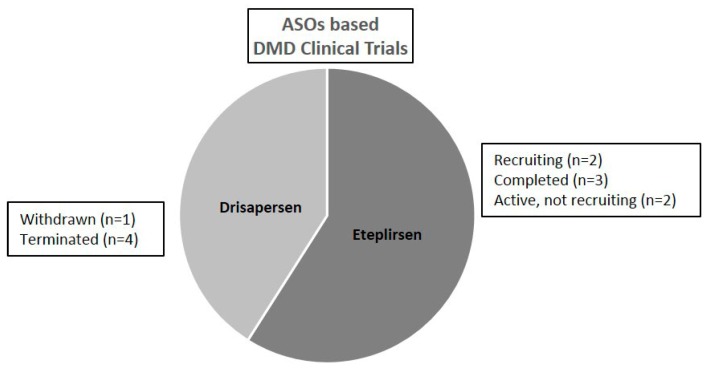
DMD antisense oligonucleotides in clinical trials. Data extrapolated from https://clinicaltrials.gov February 2017.

However, BIOMARIN received a negative feedback from FDA for Drisapersen in December 2015 [84] and shortly after from EMA. This led to the decision of BIOMARIN to stop the development of Drisapersen (as well other PS-based ASOs under either pre-clinical or clinical studies) [85]. In February 2017, on the official clinical trial website, there were reported five DMD clinical trial based on Drisapersen administration (Figure 3).

### 5.3. Morpholino ASO

Another group of chemical modified nucleic acids compounds tested in DMD clinical trials belongs to the PMO family. Their serum half-life is quite short due to the fact that as uncharged molecules they are excreted more rapidly [60]. For this reason, PMO need to be administrated at higher doses in order to reach pharmacological active concentrations. Despite the high administration doses, PMOs appear to be relatively safe as demonstrated by Sazani and collaborators with multiple repeated injections over 12 weeks of 320 mg/kg in cynomolgus monkeys [86], and the subsequent clinical studies. The first PMO ASO developed for exon 51 skipping was produced by AVI Biopharma, more recently renamed to Sarepta Therapeutics. The AVI-4658 PMO, now named Eteplirsen, was administrated by intra muscular injection into the extensor digitorum brevis muscle of 7 DMD patients [87]. Five of these 7 DMD patients received the highest dose (0.9 mg/kg) and did not report any adverse events. AVI-4658 biological efficacy was proven by the increase of mean dystrophin staining (17%) between the treated muscle compared to the contro-lateral untreated muscle. It was reported an increase of 42% of dystrophin positive fibers between the treated muscles compared to the saline injected one. Subsequently an intravenous PMO dose escalation study was undertaken on 19 DMD patients amendable to be treated for exon 51 skipping [88]. Eteplirsen was administrated from the 0.5 to 20 mg/kg as weekly intravenous infusion for 12 weeks. Muscle biopsies were collected pre and post treatment and dystrophin protein expression was analyzed by RT-PCR, immunofluorescence and Western blot. Seven patients responded to the treatment showing an increase of dystrophin fluorescence intensity from 8.9% to 16.4% compared to levels observed in the pre-treatment biopsies. Dystrophin positive fibers were counted and the high responders presented an increase of 15%, 21% and 55% compared to the number detected in pre-treatment biopsies. In the 3 high responders, Western blot was able to confirm the previous findings with an increase of protein levels after treatment from 2% to 18%, from 0.9% to 17%, and from 0% to 7.7% compared to dystrophin levels detected in healthy control muscles. Subsequently, twelve patients were recruited in a placebo controlled-phase IIb trial [89].

This study has been conceived as 24-weeks, randomized, double-blind, placebo-controlled and three different cohorts were enrolled: placebo (*n* = 4), 30 mg/kg (*n* = 4) and 50 mg/kg (*n* = 4). Pre-treatment biceps muscle biopsies were collected at baseline. The first post-treatment biopsy was scheduled after 12 weeks of administration in few patients enrolled: four from the 50 mg/kg cohort and two from the placebo subgroup. Dystrophin immunostaining in particular counting dystrophin positive fibers showed no increase between the 50 mg/kg Eteplirsen treated muscles compared to the baseline biopsies. At week 24, another subgroup of patients underwent muscle biopsy surgery (*n* = 4 of 30 mg/kg cohort and *n* = 2 of the placebo cohort). In this case, Mendell and colleagues observed an increase of 22.9% of dystrophin positive fibers in muscle sections of patients treated for 24 weeks with 30 mg/kg/week Eteplirsen regimen. After 24 weeks of double-blind treatment, the study became open-label partitioning the placebo patients in the treated cohorts (two patients per each subgroup). At week 48, a third biopsy was performed on the left deltoid in all the 12 patients enrolled.

Data from the two dose regimen cohorts (30 mg/kg and 50 mg/kg) after 48 week of Eteplirsen administration showed an increase of percentage of dystrophin-positive fibers of 47.3% compared to the pretreatment muscles. Furthermore, at 48 weeks of treatment was observed an increase of mean dystrophin intensity detected by immunofluorescence. These data, however, should be interpreted carefully when compared to clinical functional assessments mainly monitored as 6MWT performance. The adjusted mean change for the 6MWT from baseline to week 24 of the treated cohorts (30 mg/kg and 50 mg/kg) compared to the placebo subgroup are not statistically significant. At week 48, 6MWT data are statistically significant for the 50 mg/kg treated cohort but not for the 30 mg/kg patients. This is due to two patients belonging to the 30 mg/kg cohort who showed rapid and advanced disease progression. One of this patient walked 346 m at the baseline, but he declined rapidly at 112 m in the 6MWT performance assessed at week 4. This patient reported an increase of dystrophin positive fibers in the post-treatment biopsy compared to the baseline biopsy, but at the same time point of the immunofluorescence analysis he lost the ambulation [89]. This observation underlines the importance of inclusion criteria for patients enrolled in a clinical trial considering carefully the disease severity and how much muscles are already affected when patients start the treatment.

Another recent paper by Mendell and collaborators [90] presents additional functional data (6MWT and functional pulmonary tests) on the DMD treated population enrolled in the study described above. Twelve patients, previously enrolled for the 48 weeks treatment study [89], did not dismiss Eteplirsen administration and are still under treatment. Data arisen from the 6MWT have been collected semiannually from the 48 to 168 weeks of treatment. Mendell and colleagues showed that Eteplirsen treated patients present a slower 6MWT decline compared to the untreated controls. This observation could be promising for the field: indeed this DMD cohort analyzed and followed up by Mendell and colleagues is still under Eteplirsen treatment without reporting no serious adverse events and showing modest clinical benefits [90]. New assumptions in the DMD field are emerging by the analysis of clinical trial data herein reported such as the decline in the 6MWT could depend on the genetic mutation and consequent different clinical phenotype [90]. PMOs are currently developed by Sarepta Therapeutics, which is sponsoring clinical trials at different development stage: the study for skipping exon 51 (Eteplirsen-AVI4658) is currently in phase III, whereas clinical investigations for skipping exon 45 (SRP-4045) and exon 53 (SRP-4053) are currently in phase II. Currently, on the official website it has been reported that 7 DMD clinical trials are under investigations related to Eteplirsen administration (Figure 3). Sarepta submitted to FDA an accelerated approval for Eteplirsen in October 2015 and on 19 September 2016 received the conditional approval from FDA as announced on the US regulatory agency website [91] and on the biopharma company website [92]. Sarepta is also currently testing other PMO ASOs for skipping other DMD exons in pre-clinical studies.

## 6. Pre-Clinical and Clinical Approaches of ASOs in SMA

**Figure 4:**
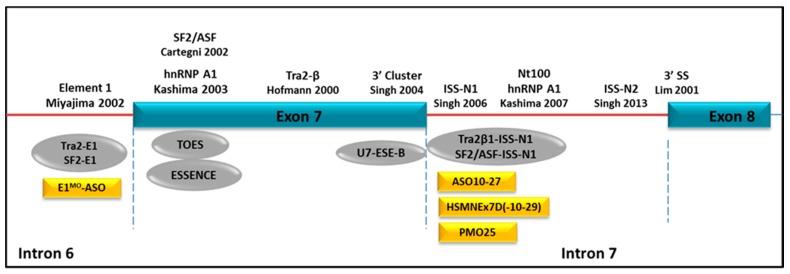
Schematic representation of *SMN2* exon 7 splicing regulatory elements and ASO targets. The functional elements regulating exon 7 splicing in *SMN2* are depicted at the top of the figure. ASOs targeting these regulatory elements are listed below, including bi-functional ASO (grey oval) and conventional ASOs (yellow rectangle).

Since *SMN2* gene is intact in all SMA patients and they have usually more than one copy of *SMN2*, alternative splicing of the *SMN2* gene has become an attractive therapeutic target by strategies to augment the inclusion of exon 7 in the mature transcript. In the *SMN2* gene, a few regulatory elements have been shown to modulate the alternative splicing of exon 7. These include element 1 in intron 6, an SF2/ASF or hnRNP A1 binding site in exon 7, the Tra2-β1 binding site in exon 7, the 3′ cluster at the 3′-end of exon 7, the splicing silencer N1 (ISS-N1) and a recently reported ISS-N2 in intron 7, and hnRNP A1 binding sites in intron 7 (Figure 4) [22,23,93,94,95,96,97,98,99].

Two approaches have emerged for stimulating the inclusion of *SMN2* exon 7: (i) the use of ASOs to block silencer motifs, e.g., exonic splicing silencers (ESSs) or intronic splicing silencers (ISSs); or (ii) the use of bi-functional ASOs to provide trans-acting exonic splicing enhancers (ESEs) in exon 7. The bi-functional ASOs were designed with one domain that was intended to anneal to the target exon and a second (tail) domain that contained sequences (i.e., the GGA motif) to which activator proteins, such as the SR proteins, would bind. A method termed ‘targeted oligonucleotide enhancers of splicing (TOES)’ has been described utilizing such strategy. TOES stimulated the splicing of *SMN2* exon 7 in nuclear extracts of a cell-free model and in fibroblasts derived from SMA patients [100,101]. A similar strategy, “exon-specific silencing enhancement by small chimeric effectors” (ESSENCE), which comprises a minimal synthetic RS domain to emulate the function of SR proteins, has also shown efficacy in redirecting exon 7 splicing in vitro [102]. In contrast to the promising result in vitro, in vivo evaluation of bi-functional ASOs in SMA mice showed far less efficacy than those from conventional ASOs [103]. A bi-functional ASO targeting the intronic repressor element 1 (E1) in intron 6 showed quite modest efficacy in SMA mice in terms of phenotype rescue [103]. However, subsequent studies using conventional ASOs to target the intronic splicing silencer N1 (ISS-N1) sequence in intron 7 has remarkably boosted the investigation of using ASOs as therapeutic strategies in SMA, as they all displayed striking efficacy in rescuing SMA mice in different transgenic mouse models with different chemical modifications of ASOs.

ISS-N1 is a 15 nucleotides sequence starting from the +10 to +24 nucleotide position in *SMN2* intron 7. Its function in regulating exon 7 inclusion in *SMN2* gene was first reported by Singh et al. in 2006 and was further confirmed by Hua et al. in 2008 [97,104]. A considerable number of in vivo studies in SMA transgenic mice using ASOs targeting ISS-N1 sequence have since conducted and reported the most encouraging data so far in SMA experimental therapy. In these studies, three different chemical modifications in ISS-N1 ASOs have been tested. These ASOs chemistries include 2′-OMe, *2′-O-2-*methoxyethyl phosphorothioate (MOE) and PMO ASOs. The first ISS-N1 ASO study was conducted by the Krainer group using MOE and intravenously injected in adult unaffected heterozygous hSMN2 transgenic mice. While significant correction of exon 7 splicing was observed in peripheral tissues, e.g., in liver, kidney and muscle, there was no correction detected in spinal cord [104]. This result was expected, as MOEs do not penetrate the BBB. Direct CNS delivery of ISS-N1 ASO on a 2′-OMe backbone, was hence reported in SMA transgenic mice. Although there was significant enhancement of SMN protein throughout the CNS, which was accompanied by an increase in bodyweight, after repeated intracerebroventricular (ICV) injections in neonatal Δ7SMA mice, there was unfortunately no data reported on survival when all experiments ended at P12 [105]. Furthermore, a pro-inflammatory effect in the brain and the spinal cord was detected when the 2′-OMe ASO was administered into the CNS, in the absence of any effect on *SMN2* exon 7 inclusion [106]. Modification of the backbone to MOE chemistry, by using an 18-mer ISS-N1 ASO (ASO-10-27), significantly extended the lifespan of SMA mice after systemic administration [26]. Further in vivo studies of a number of ASOs on PMO backbone, by targeting the ISS-N1 motif and its flanking sequence, have also shown the striking rescue to the severe SMA transgenic mouse models [28,29,30]. There is length-dependent effect of PMO ASO [29]. The superiority of the 25 mer PMO (−10, −34) to the shorter 20 or 18 mer PMOs, on augmenting SMN2 exon 7 splicing in cellular assay and tissues as well as on improving survivals in SMA mouse models has been confirmed in three independent studies from different laboratories [29,30,107,108]. In addition, the combined systemic and local ICV PMO treatment is suggested to be more beneficial in SMNΔ7 mice than individual respective delivery route [108]. In a suboptimal dose of 25 mer PMO generated intermediate SMA mice, the second administration of PMO by systemic injection significantly increased the survival and improved the phenotypes in comparison to ICV injections [107].

Clinical trials for one of these compounds have rapidly moved from phase I, to phase II, phase III studies in infants with type I SMA, and older non-ambulant children with SMA II and III. This ASO, named Nusinersen (IONIS-SMNRX, ASO-10-27), was initiated in children with SMA by Ionis pharmaceutics in 2012 (ClinicalTrials.gov ID: NCT01494701) (http://clinicaltrials.gov). In the first open-label and escalating dose study reported in Table 1, patients tolerated well a single dose treatment of 1, 3, 6, or 9 mg ASO administered intrathecally by lumbar puncture. Moreover, at highest dose of 9 mg, patient’s functional abilities significantly improved at 3 months post dose and further increased 9–14 months post dose during the extension study [8]. With this encouraging data, a following phase Ib/IIa has tested the safety, tolerability and pharmacokinetics of escalating multiple doses of Nusinersen administered intrathecally (ClinicalTrials.gov ID: NCT01703988) to SMA patients aged 2 to 15 years old.

Finally, a large phase III multicenter trial for infants SMA was also started by Ionis to assess the efficacy and safety of Nusinersen in infants with SMA (ClinicalTrials.gov ID: NCT02193074). Remarkably, this latter study was recently interrupted early as the study had met a pre-specified functional primary endpoints and currently all children are being transitioned from the placebo controlled part of the study to an open label in which all children receive the active treatment.

In December 2016, FDA announced the approval of Nusinersen, with the commercial name of Spinraza, as first drug approved for treating SMA [109]. Spinraza is now available on the market from Biogen [110]. SMA has traditionally been considered as a selective lower motor neuron disease with spinal motor neurons in the anterior horn being the primary pathological target. Increasing numbers of clinical and experimental studies indicate the involvement of additional peripheral organs, such as cardiac dysfunctions and distal digits necrosis, in the pathogenesis of the disease progression, especially in cases at the severe end of the clinical spectrum [111,112]. The spectrum of affected organs is even wider in the severe SMA mouse models, with the involvement of muscle, brain, heart, afferent nerves, vasculature, bone, pancreas, liver, lung and even the reproductive system [112,113,114,115,116,117,118]. The combined CNS and systemic administration of ASOs achieves the best survival rates in the SMA transgenic mice [107,108]. It has been demonstrated that regular systemic administration of PMO ASO in an intermediate SMA mouse model from birth until adulthood provided therapeutic benefit and rescued the SMA phenotype, presumably via peripheral SMN restoration [107]. SMN is critical for motor neurons development and neuromuscular junction maturation [119,120] and complete loss of SMN is embryonic lethal and results in the embryo’s death prior to implantation [121,122].

On the other hand, the report of the post-symptomatic rescue by systemic ASO treatment in SMA mice indicates that the effect is likely due to strengthening of the existing NMJs more than on their maturation [123]. SMN restoration by ASO in peripheral organs may provide further therapeutic benefit and phenotypic rescue, at least in mouse. However, the relevance of the peripheral organ pathology for disease progression in the human is currently unknown. . The recent positive clinical trial data from Ionis clearly suggests that restoring SMN protein expression in the CNS is sufficient to induce a significant benefit to infants with type I SMA. Whether there will be an advantage to also restore SMN in the periphery remains a question for future investigations.

## 7. ASO Therapy in Other Neuromuscular Disorders

In recent years, the antisense oligonucleotide strategy has been evaluated for the treatment of many other neuromuscular diseases such as myotonic dystrophy, oculopharyngeal muscular dystrophy, laminopathies, Pompe disease, congenital myasthenic syndromes and facioscapulohumeral muscular dystrophy [124]. Promising results have been obtained from pre-clinical studies and herein we report two additional examples of applying ASO strategies in the neuromuscular field.

### 7.1. Myotonic Dystrophy

Myotonic Dystrophy (DM) is one of the most common neuromuscular disorders in adults. There are two genetically distinct forms, Myotonic dystrophy type 1 (DM1, Steinert’s disease) caused by CTG repeat expansion in the 3′ UTR region of the myotonic dystrophy protein kinase (*DMPK*) gene, and Myotonic dystrophy type 2 (DM2, proximal myotonic myopathy) due to CCTG repeats in the intron 1 of the *CNBP* gene (also known as CCHC-type zinc finger, nucleic acid binding protein). At the molecular level, both mutations result in a toxic gain-of-function of the mutated RNA that is retained in the nuclei as aggregates or foci affecting the function of alternative splicing regulators such as MBNL and CELF1 proteins [125]. At present, there is no treatment for this disease even though different therapeutic approaches targeting both RNA and protein have been evaluated [124]. In preclinical studies, Wheeler and collaborators developed a 2′-*O*-methoxyethyl (MOE) gapmer ASO able to induce the degradation of target mutant RNA transcripts through the RNAase-H mechanism. They demonstrated that the MOE gapmer-ASO was active against the mutant *DMPK* mRNA with a reduction both in vivo and in vitro of the aberrant transcript [125,126]. These results supported the first DM1 clinical trial, initiated by Ionis Pharmaceuticals, based on antisense oligonucleotide strategies (ISIS-DMPKRx). Recently, the phase I/IIa clinical trial conducted in DM1 adult patients was completed by Ionis [127]. This dose escalation study assessed the safety and tolerability of the ASO targeting mutant *DMPK* transcript. Muscle biopsies were collected and primary biological endpoints as biomarker monitoring and splicing changes were evaluated. Ionis reported small, but not significant, biological changes in muscle biopsies of patients and observed that the ASO concentration was not reaching the therapeutic dose to support potential clinical benefits. Therefore, Ionis decided to stop the clinical trial and to expand the pre-clinical development of LICA (Ligand-Conjugated Antisense) molecules for treating myotonic dystrophy [127].

### 7.2. Facioscapulohumeral Muscular Dystrophy

Facioscapulohumeral muscular dystrophy (FSHD) is one of the most prevalent adult muscular dystrophies, characterized by weakness affecting the muscles of the face, shoulder, and arms. About 95% of patients (classified as FSHD1) present deletions of repeated sequence in the D4Z4 region on chromosome 4q35.3 and a subpopulation (5%) of cases (named as FSHD2) have mutations that exert their effect by D4Z4 deletion-independent pathway [128,129]. Different candidate genes have been explored and recently the de-repression of the retrogene *DUX4* is believed to cause the pathology in FSHD in a toxic-gain-of-function manner [130]. Therefore, the inhibition of aberrant *DUX4* expression by gene silencing could be a promising therapeutic approach. Two groups, Marsollier et al. and Chen et al. reported the efficacy of antisense phosphorodiamidate morpholino oligonucleotide to suppress the expression of *DUX4* in both a FSHD cell model [71,72] and FSHD patient muscle xenografts in mice [72] by targeting the polyadenylation signal of *DUX4*. They also demonstrated that the PMO can decrease the abnormally high expression of many genes downstream of DUX4 pathway. These results demonstrate the potential relevance of using PMO for treating FSHD.

## 8. Delivery of ASOs

One of the major issues for the use of ASOs for therapeutic purposes is the efficient delivery to the target site. DNA and RNA molecules need to cross several physiological barriers before reaching their intracellular targets, in particular, the tissue and cellular barriers [131].

After administration, the ASOs should reach the target tissue without affecting other organs, avoiding their rapid degradation in biological fluids and fast clearance. In addition, they should cross the vascular endothelial barrier and enter into the right intracellular compartment [7,132]. Since the chemical and physical properties of ASO compounds have an impact on their pharmacokinetics and biodistribution, extensive studies to modify the chemical structures in order to improve their stability, specificity and to reduce the toxicity have been performed, even if more investigations are needed to improve further cellular uptake and tissue targeting [133]. Two main strategies can be used for ASO delivery: viral and non-viral delivery.

Despite viral vectors are efficient systems for the delivery of genetic material and for the capability to infect a large number of cell types, they showed some limitations for clinical applications. These constraints are: (i) the immunogenicity which results in poor retention within the cells and induction of inflammatory reactions; (ii) tumorigenicity risks; (iii) their limited loading capacity and (iv) scaling-up problems [131,134]. Non-viral delivery represents a good alternative due to the absence of immune response [135], the stability, the possibility to bind high quantity of nucleic acid, and to perform surface modification for targeting specific tissues [131].

The conjugation of free ASOs with non-viral delivery carriers can be achieved by two approaches, one is to incorporate them into nanoparticles (NPs) that, based on their size and materials, will determine the ASO biodistribution and interaction; the second is to conjugate the ASO with a targeting ligand, that can recognize specific receptors and enter in the cells via receptor-mediated endocytosis [7,132].

The main difference between the two methods is the size, nanoscale for the first and molecular scale for the latter, and both approaches present different advantages and drawbacks. Due to their sizes, the NPs have a limited biodistribution because they cannot cross the capillary endothelium. Conversely, conjugate complexes show a broad biodistribution since they can pass efficiently the capillary endothelium. However, due to the small size, they have a rapid renal clearance. Nanoparticles can bind large amount of oligonucleotides that enter into the cells in a single event, but in order to reach the effective ASO concentration a high number of nanomaterials should be delivered and they could accumulate with a potential toxicity, especially if nanoparticles are biocompatible but no biodegradable. Instead, molecular conjugates are able to delivery only a single oligonucleotide for each uptake, and numerous events are required to obtain a pharmacological concentration. Furthermore, the NPs-ASO complex can protect the nucleic acid from nucleases, unlike the conjugates [7].

Different studies to increase the cellular delivery of therapeutic ASOs for RNA splicing regulation were performed using the arginine-rich (CPPs). As already mentioned in Section 2.2, the most successful CPP conjugation is with ASO species neutrally charged as PNA and PMO [46]. This approach was tested in preclinical DMD and SMA studies, and *in vivo* analyses were performed in both *mdx* and SMA mice.

Wu and colleagues tested the administration of a CPP conjugated PMO in *mdx* mice and demonstrated the restoration of dystrophin in skeletal muscles and interestingly also in the heart [136]. Then, a series of peptide nucleic acid/ PMO internalization peptides (Pips) derived from CPP Penatratin were tested for their higher stability to serum proteolysis. Pip2a and Pip2b peptides were able to induce exon skipping into dystrophin transcript after intramuscular injection in *mdx* mice [50]. Modifications of Pips compounds led to identify an alternative CPP conjugated ASO (Pip5e-PMO) with a better dystrophin splicing modulation activity after systemic administration in mdx mice, including in the heart [54].

However, positively charged peptides increase the toxicity, making them not useful for lifelong administration like in DMD [137].

Pip-PMO conjugated has also been investigated in preclinical SMA studies. A pip6a-PMO has recently been examined in SMA mice and showed body-wide SMN restoration in both peripheral and CNS tissues after systemic administration [138]. The successful rescue of SMA phenotype and the dramatic prolongation in lifespan of the severe SMA mice demonstrates the high potential of Pip-PMO therapy for SMA [138]. The (RXR)_4_ peptide, B-peptide or phage peptides showed to enhance the in vivo exon skipping activity in respect to the naked ASO, also in the cardiac muscle [133].

Another class of conjugates is the vivo-morpholinos (vPMOs), dendrimeric octaguanidines oligomers able to modulate efficiently the splicing [139]. However, no improved therapeutic efficacy was observed in vivo in the severe SMA mouse models when vivo-morpholino was compared to naked PMO which is likely due to the potential toxicity in vivo [29,108].

Wang and collaborators evaluated for the first time the cationic polyelectrolytes (PEs) as vectors for PMO in in vitro and in vivo (*mdx* mice) experiments. They demonstrated that the poly (diallyldimethylammonium chloride) (PDDAC) polymer series can improve the delivery of PMO without local toxicity [140].

In recent years, the progress of nanotechnology has provided several nanosystems with the aim to increase the drug targeting efficacy. Based on the material, the nanoparticles can be classified in different categories and the most common types used for drug delivery are:

- Solid Lipid Nanoparticles (SLNs) that, made of natural, semi-synthetic or synthetic lipids, are biocompatible and produced easily at large scale systems, can protect drugs from degradation and control the release of the molecules [141].
- Polymer nanoparticles (including micelles, nanocapsules, nanospheres, colloids, dendrimers, core-shells) in which the drug can be loaded by different methods, i.e., entrapment, dispersion, dissolution, or adsorption [142]. Examples of polymer nanoparticles used to conjugate ASOs for exon skipping application in the DMD field are cationic core-shell NPs, named T1 and ZM2, made up of a polymethyl methacrylate (PMMA) core surrounded by a cationic shell for the ASO binding. Both T1 and ZM2 NPs showed the capability to delivery 2′OMePS M23D ASO in *mdx* mice. In particular, intraperitoneal and oral administrations of ZM2-ASOs complexes induced dystrophin restoration in preclinical studies [143,144,145,146,147].
- Lipid-based Nanoparticles (LNPs), composed of cationic lipid and other compounds called “helper lipids” that contribute to their stability and delivery efficiency. LNPs for antisense delivery usually encapsulate the ASO inside the aqueous core and between bilayers [148].
- Carbon-based nanomaterials, like graphene, nanodiamond, fullerenes, nanotubes, are used for various applications including medicine for drug delivery and imaging [149].

All the delivery systems discussed above, together with other available vectors not presented here, can be used in other diseases where ASO treatment is an eligible therapeutic approach such as SMA and myotonic dystrophy.

Generally, biocompatible nanoparticles are not biodegradable and tend to accumulate especially if repeated doses are administered, as in chronic diseases. Therefore, fully biodegradable nanoparticles, as nanomolecules of albumin, exosomes, or nanodroplets are much more appealing to deliver drugs, including ASOs.

Further chemical modifications of ASOs and the development of these new carriers for optimizing their delivery could improve the efficacy of such treatments.

## 9. Conclusions

In the last two decades, there has been remarkable progress in the molecular understanding of the common neuromuscular conditions, which has resulted in an impressive improvement in the diagnosis and management for these conditions. In the last few years, this knowledge has been also exploited to develop novel innovative therapeutic interventions, in which ASOs played a central role.

Indeed, the human genome project output greatly contributed to these goals, and made feasible new therapeutic approaches as classical gene therapy, splicing modulation, RNA interference. It is likely that the combination of these strategies will expand even further for the treatment of rare neuromuscular (and other) disorders. In addition, the revolutionary CRISP-Cas9 genome editing may offer more novel therapeutic opportunities [150], if safety issues and off-target effects are solved. ASO therapies remain a valid and relatively safe approach, with increased use in clinical trials and now either approved, or in the path for approval, for several disorders. Delivery of ASOs is still a key point that should be carefully addressed, since tissue targeting is vital to reach satisfactory drug distribution therefore increasing therapy efficiency.

Preclinical and clinical investigations for treating neuromuscular disorders have been at the cutting edge on developing extraordinarily gene therapy studies. 

We anticipate that ASO approaches for treating severe neuromuscular conditions will be a research model for improving gene therapy investigations for other disorders. Furthermore, current investigations in this field will enhance ASOs delivery improving tissue targeting specificity and increasing ASO efficacy.

## Figures and Tables

**Table 1:** Summary table of clinical trials based on ASO therapy for treating neuromuscular disorders. DMD, Duchenne Muscular Dystrophy; PS, phosphorothioate; 2′OMe, 2′-O′methyl; PMO, phosphorodiamidate morpholino; SMA, Spinal Muscular Atrophy; MOE, 2′-*O*-methoxyethyl. (*) indicates that the same patient group was monitored in both the two studies.

Disease	ASO	Number of Patients	Reference
DMD			
	PS	1	[77]
	2′OMe (Drisarpersen)	4	[78]
	2′OMe (Drisarpersen)	12	[79]
	2′OMe (Drisarpersen)	53	[81]
	PMO (Eteplirsen)	7	[87]
	PMO (Eteplirsen)	19	[88]
	PMO (Eteplirsen)	12 (*)	[89]
	PMO (Eteplirsen)	12 (*)	[90]
SMA			
	2′MOE (Nusinersen)	28	[8]
